# Pre-hospital application of REBOA for life-threatening hemorrhage

**DOI:** 10.1186/s40779-023-00504-5

**Published:** 2023-12-13

**Authors:** Xiao-Mei Tian, Wei Hu, Feng-Yong Liu

**Affiliations:** 1 Department of Interventional Radiology, Senior Department of Oncology, the Fifth Medical Center of Chinese PLA General Hospital, Beijing, 100039 China; 2 Medical School of Chinese PLA, Beijing, 100853 China; 3 Department of Emergency, the Fifth Medical Center of Chinese PLA General Hospital, Beijing, 100039 China; 4 Senior Department of Infectious Diseases, the Fifth Medical Center of Chinese PLA General Hospital, National Clinical Research Center for Infectious Diseases, Beijing, 100039 China

**Keywords:** Resuscitative endovascular balloon occlusion of the aorta (REBOA), Pre-hospital, Endovascular, Aortic balloon occlusion, Trauma, Hemorrhage, Shock

Dear Editor,

Most battlefield casualties occur prior to the arrival of medical facilities. Uncontrollable hemorrhage accounts for more than 90% of those potentially survivable battlefield casualties [1]. In both military and civilian conditions, non-compressible torso hemorrhage always caused rapid exsanguination and high mortality rates before definitive treatment [2]. More than half of the deaths due to non-compressible torso hemorrhage occur before hospital care can be provided [2]. Therefore, early and rapid pre-hospital hemorrhage control is essential to reduce mortality.

Resuscitative endovascular balloon occlusion of the aorta (REBOA) is a life-saving procedure for patients with non-compressible hemorrhage and severe hemorrhagic shock [3]. In addition to in-hospital REBOA, urgent REBOA can be rapidly completed in grim pre-hospital situations for patients [4]. Thus, pre-hospital REBOA application for the treatment of life-threatening hemorrhages has attracted increasing attention. In patients who receive timely pre-hospital REBOA treatment, the mortality can be reduced to less than 40% [5]. In this letter, we focus on the pre-hospital application of REBOA for managing life-threatening traumatic hemorrhages in both military and civilian settings.

REBOA was first introduced by the US Army in the Korean War to treat intraabdominal hemorrhages. With significant improvements in endovascular equipment and techniques, pre-hospital REBOA has attracted renewed clinical interest. Recently, the US Army reported the use of pre-hospital REBOA in treating modern combat casualties [6]. After pre-hospital REBOA treatment, the patients were finally hemodynamically stabilized and safely evacuated without any apparent complications. Furthermore, the Russian Army have also validated the effectiveness of pre-hospital REBOA on the battlefield [7]. When combined with other resuscitation strategies like blood transfusion, pre-hospital REBOA can further enhance survival rates. Therefore, it is evident that pre-hospital REBOA is an effective method for acute care of massive hemorrhage and can be safely performed in the battlefield setting as an emergency treatment option for individuals at risk of cardiovascular failure due to injuries sustained in combat situations. On the battlefield, frontline implementation of REBOA allows temporary hemorrhage control and facilitates timely evacuation to the hospital, thereby reducing mortality rate and improving overall treatment outcomes, simultaneously saving lives among military personnel. This technology is of great significance for military applications and may become an essential skill for military training programs and medical practices in the future.

In addition to the battlefield environment, pre-hospital REBOA is also suitable for trauma patients in civilian conditions. Uncontrolled hemorrhagic shock or cardiac arrest accounts for a significant percentage of trauma patients. Some of these patients could benefit from pre-hospital REBOA. For patients severely injured from a high drop, REBOA serves as a safe and effective surgical technique that reduces blood loss and stabilizes the patient’s hemodynamic state, allows for longer transport times, and provides an opportunity for definitive hemostasis [8]. Pre-hospital REBOA uses a portable balloon catheter device to perform emergency bleeding control at the scene, rapidly controlling bleeding, maintaining hemodynamic stability, and providing resuscitative support to avoid death from hemorrhagic shock. Postoperative complications such as lower limb ischemia, organ ischemia–reperfusion injury, and aortic dissection occur less frequently. In the future, further standardized training platforms are needed to guide physicians to familiarize themselves with REBOA technology, indications, adverse reactions, and postoperative care.

In contrast to in-hospital conditions, REBOA performed in the grim pre-hospital environment requires a coordinated emergency health care system with a well-trained and -equipped care team. Given that battlefield or pre-hospital casualties often require evacuation, it is crucial to ensure the establishment of arterial access and successful placement of the arterial sheath. Successful REBOA placement has been reported on a rotary-wing platform [4]. Thus, it is recommended that eligible patients are promptly evacuated while performing REBOA en route during the evacuation period.

The prompt implementation of REBOA is crucial in the pre-hospital management of traumatic hemorrhage, emphasizing the necessity for expeditious surgical intervention. Previously, prior to initiating REBOA, ultrasonic or digital subtraction angiography should be performed. With improvements in catheterization devices and techniques, the competencies and successful rates of blind performance increased significantly, especially after comprehensive REBOA training. Following team training, the average time to complete the pre-hospital REBOA procedure under ultrasound guidance was 8.5 min [9]. Thus, standardized training and ample experience is imperative to ensure competency in pre-hospital REBOA. Given its favorable success rate, pre-hospital REBOA holds great appeal for both military and civilian conditions.

In conclusion, pre-hospital REBOA demonstrates promising potential as a temporary and minimally invasive bridge approach for eventual control of non-compressible traumatic hemorrhage. By occluding the aorta, REBOA effectively redistributes cardiac output to vital organs. As revealed so far, pre-hospital REBOA has shown favorable outcomes in controlling non-compressible torso hemorrhage (Fig. 1). The implementation of REBOA and its clinical training courses will improve the efficacy of pre-hospital treatment for life-threatening traumatic hemorrhages, ultimately reducing the loss of life caused by traumatic hemorrhage.

**Fig. 1:**
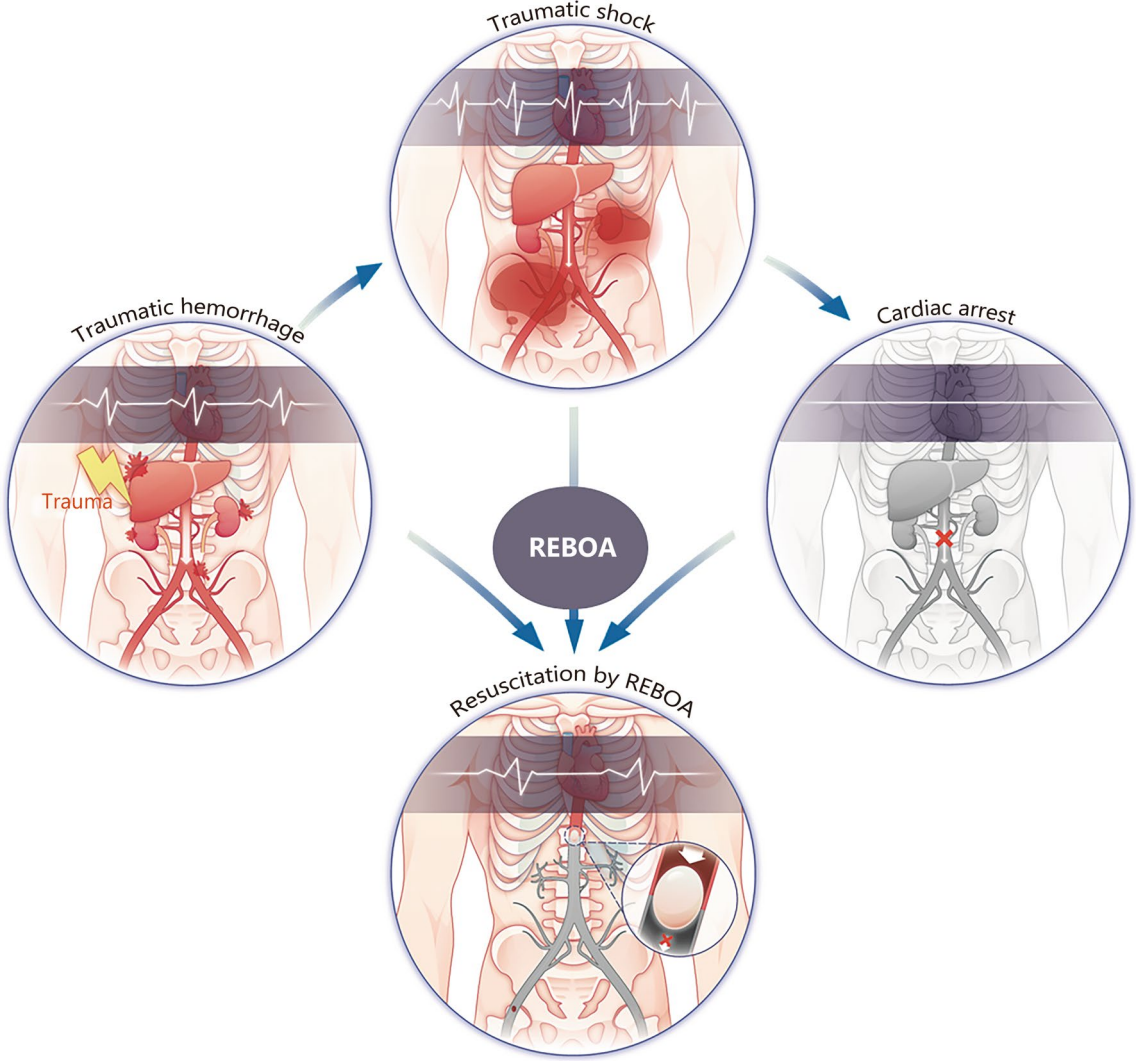
Pre-hospital application of resuscitative endovascular balloon occlusion of the aorta (REBOA) for life-threatening hemorrhage. Non-compressible traumatic hemorrhage could result in massive bleeding and hemorrhage shock. Pre-hospital utilization of REBOA could effectively stop persistent bleeding and prevent hemorrhage shock

## Data Availability

Not applicable.

## Abbreviation

REBOA: Resuscitative endovascular balloon occlusion of the aorta
